# Supplementary material for: Modeling glioblastoma heterogeneity as a dynamic network of cell states
Source: Mol Syst Biol. 2021 Sep 16;17(9):e10105. doi: 10.15252/msb.202010105 (PMC8444284; doi:10.15252/msb.202010105)
Supplement: Supplementary file 5 — Source Data for Figure 3 [file MSB-17-e10105-s001.zip › Figure3A_sourcedata/GSEA_3065/nosigdb_state3.GseaPreranked.1622718426028/gsea_report_for_na_neg_1622718426028.html]

Report for na\_neg 1622718426028 [GSEA]

| GS  follow link to MSigDB | GS DETAILS | SIZE | ES | NES | NOM p-val | FDR q-val | FWER p-val | RANK AT MAX | LEADING EDGE || 1 | LARSSON\_STATE\_1 | Details ... | 97 | -0.83 | -2.92 | 0.000 | 0.000 | 0.000 | 695 | tags=70%, list=8%, signal=75% |
| 2 | LARSSON\_STATE\_6 | Details ... | 98 | -0.80 | -2.86 | 0.000 | 0.000 | 0.000 | 850 | tags=66%, list=10%, signal=73% |
| 3 | NB\_KI\_CLUSTER1 | Details ... | 32 | -0.95 | -2.80 | 0.000 | 0.000 | 0.000 | 327 | tags=97%, list=4%, signal=100% |
| 4 | OLSEN\_ADRENERGIC | Details ... | 62 | -0.66 | -2.20 | 0.000 | 0.000 | 0.000 | 945 | tags=58%, list=11%, signal=65% |
| 5 | RICHARDS\_NOWAKOWSKI\_SCIENCE\_2017\_IPC\_NEN3\_UPREG | Details ... | 224 | -0.52 | -2.06 | 0.000 | 0.000 | 0.001 | 663 | tags=23%, list=8%, signal=24% |
| 6 | RICHARDS\_NOWAKOWSKI\_SCIENCE\_2017\_RG\_EARLY\_UPREG | Details ... | 426 | -0.48 | -2.00 | 0.000 | 0.000 | 0.003 | 1221 | tags=31%, list=14%, signal=34% |
| 7 | NB\_KI\_CLUSTER3 | Details ... | 160 | -0.49 | -1.88 | 0.000 | 0.003 | 0.030 | 1192 | tags=33%, list=14%, signal=37% |
| 8 | RICHARDS\_NOWAKOWSKI\_SCIENCE\_2017\_MGE\_RG2\_UPREG | Details ... | 434 | -0.42 | -1.78 | 0.000 | 0.006 | 0.087 | 1027 | tags=26%, list=12%, signal=28% |
| 9 | NB\_KI\_CLUSTER4 | Details ... | 89 | -0.50 | -1.74 | 0.000 | 0.009 | 0.130 | 1450 | tags=48%, list=17%, signal=57% |
| 10 | RICHARDS\_VERHAAK\_CANCERCELL\_2010\_NEURAL | Details ... | 56 | -0.52 | -1.73 | 0.008 | 0.009 | 0.145 | 2034 | tags=36%, list=23%, signal=46% |
| 11 | OLSEN\_MESENCHYMAL | Details ... | 68 | -0.48 | -1.63 | 0.005 | 0.026 | 0.390 | 263 | tags=21%, list=3%, signal=21% |
| 12 | FELDMAN\_2020\_NEURAL G0 | Details ... | 363 | -0.39 | -1.60 | 0.000 | 0.032 | 0.481 | 1364 | tags=39%, list=16%, signal=45% |
| 13 | IVY\_IT | Details ... | 31 | -0.55 | -1.59 | 0.007 | 0.033 | 0.521 | 1830 | tags=52%, list=21%, signal=65% |
| 14 | SUVA\_MES1\_PMID\_31327527 | Details ... | 38 | -0.53 | -1.57 | 0.013 | 0.035 | 0.571 | 547 | tags=26%, list=6%, signal=28% |
| 15 | RICHARDS\_NEFTEL\_CELL\_2019\_MES1 | Details ... | 38 | -0.53 | -1.57 | 0.015 | 0.034 | 0.592 | 547 | tags=26%, list=6%, signal=28% |
| 16 | FELDMAN\_2020\_S-G2 | Details ... | 323 | -0.38 | -1.57 | 0.000 | 0.033 | 0.603 | 1361 | tags=36%, list=16%, signal=41% |
| 17 | RICHARDS\_NOWAKOWSKI\_SCIENCE\_2017\_NIN4\_UPREG | Details ... | 130 | -0.40 | -1.50 | 0.006 | 0.054 | 0.800 | 967 | tags=22%, list=11%, signal=24% |
| 18 | NB\_KI\_CLUSTER11 | Details ... | 49 | -0.47 | -1.49 | 0.030 | 0.057 | 0.841 | 1426 | tags=39%, list=16%, signal=46% |
| 19 | DOROSZKO\_U3180MG\_CLUSTER\_4 | Details ... | 161 | -0.39 | -1.47 | 0.004 | 0.062 | 0.889 | 1144 | tags=28%, list=13%, signal=32% |
| 20 | GAROFANO\_IAVARONE\_MTC\_SUBTYPE | Details ... | 207 | -0.38 | -1.47 | 0.004 | 0.061 | 0.898 | 916 | tags=16%, list=10%, signal=18% |
| 21 | RICHARDS\_NOWAKOWSKI\_SCIENCE\_2017\_IPC\_NEN1\_UPREG\_1 |  | 129 | -0.40 | -1.47 | 0.009 | 0.059 | 0.904 | 465 | tags=16%, list=5%, signal=17% |
| 22 | RICHARDS\_NOWAKOWSKI\_SCIENCE\_2017\_MGE\_IPC3\_UPREG |  | 112 | -0.40 | -1.45 | 0.016 | 0.064 | 0.932 | 364 | tags=17%, list=4%, signal=17% |
| 23 | DOROSZKO\_U3180MG\_CLUSTER\_5 |  | 31 | -0.50 | -1.43 | 0.058 | 0.071 | 0.948 | 985 | tags=42%, list=11%, signal=47% |
| 24 | DOROSZKO\_U3013MG\_CLUSTER\_3 |  | 97 | -0.41 | -1.43 | 0.021 | 0.070 | 0.954 | 1293 | tags=38%, list=15%, signal=44% |
| 25 | FELDMAN\_2020\_G2-M |  | 278 | -0.35 | -1.41 | 0.009 | 0.078 | 0.972 | 1221 | tags=28%, list=14%, signal=32% |
| 26 | RICHARDS\_NOWAKOWSKI\_SCIENCE\_2017\_NEN\_LATE\_UPREG |  | 349 | -0.34 | -1.40 | 0.000 | 0.086 | 0.983 | 1066 | tags=19%, list=12%, signal=21% |
| 27 | RICHARDS\_NOWAKOWSKI\_SCIENCE\_2017\_IN\_CTX\_CGE1\_UPREG |  | 292 | -0.34 | -1.37 | 0.012 | 0.099 | 0.992 | 1079 | tags=20%, list=12%, signal=22% |
| 28 | FELDMAN\_2020\_M-EARLYG1 |  | 105 | -0.38 | -1.37 | 0.033 | 0.103 | 0.994 | 1018 | tags=32%, list=12%, signal=36% |
| 29 | RICHARDS\_NEFTEL\_CELL\_2019\_MES2 |  | 42 | -0.44 | -1.35 | 0.074 | 0.116 | 0.996 | 709 | tags=19%, list=8%, signal=21% |
| 30 | SUVA\_MES2\_PMID\_31327527 |  | 42 | -0.44 | -1.33 | 0.101 | 0.127 | 0.997 | 709 | tags=19%, list=8%, signal=21% |
| 31 | LARSSON\_STATE\_4 |  | 100 | -0.37 | -1.32 | 0.067 | 0.134 | 0.998 | 1208 | tags=36%, list=14%, signal=41% |
| 32 | GANDAL\_ALCOHOLADDICTION\_UP\_PMID29439242 |  | 136 | -0.33 | -1.23 | 0.085 | 0.244 | 1.000 | 1227 | tags=22%, list=14%, signal=25% |
| 33 | NB\_KI\_CLUSTER16 |  | 70 | -0.36 | -1.21 | 0.158 | 0.259 | 1.000 | 1379 | tags=27%, list=16%, signal=32% |
| 34 | SINGH\_SOX2\_DOWNREGULATED\_TRANSCRIPTS\_PMID\_28122245 |  | 84 | -0.34 | -1.21 | 0.134 | 0.252 | 1.000 | 1306 | tags=21%, list=15%, signal=25% |
| 35 | DOROSZKO\_U3180MG\_CLUSTER\_3 |  | 466 | -0.29 | -1.20 | 0.059 | 0.261 | 1.000 | 1362 | tags=24%, list=16%, signal=27% |
| 36 | RICHARDS\_NOWAKOWSKI\_SCIENCE\_2017\_IN\_CTX\_MGE1\_UPREG |  | 271 | -0.29 | -1.18 | 0.102 | 0.292 | 1.000 | 1149 | tags=17%, list=13%, signal=19% |
| 37 | FELDMAN\_2020\_S |  | 149 | -0.31 | -1.17 | 0.154 | 0.300 | 1.000 | 1297 | tags=29%, list=15%, signal=33% |
| 38 | RICHARDS\_NOWAKOWSKI\_SCIENCE\_2017\_IN\_CTX\_MGE2\_UPREG |  | 190 | -0.30 | -1.17 | 0.155 | 0.297 | 1.000 | 847 | tags=16%, list=10%, signal=18% |
| 39 | RICHARDS\_NOWAKOWSKI\_SCIENCE\_2017\_EN\_PFC3\_UPREG |  | 190 | -0.30 | -1.17 | 0.150 | 0.293 | 1.000 | 635 | tags=12%, list=7%, signal=13% |
| 40 | RICHARDS\_NEFTEL\_CELL\_2019\_NPC2 |  | 32 | -0.40 | -1.16 | 0.247 | 0.299 | 1.000 | 1574 | tags=38%, list=18%, signal=46% |
| 41 | SUVA\_NPC2\_PMID\_31327527 |  | 32 | -0.40 | -1.14 | 0.276 | 0.333 | 1.000 | 1574 | tags=38%, list=18%, signal=46% |
| 42 | WANG\_MESENCHYMAL\_PMID\_28697342 |  | 23 | -0.42 | -1.14 | 0.285 | 0.329 | 1.000 | 775 | tags=17%, list=9%, signal=19% |
| 43 | DALMO\_EXTINCTION\_UNPUB |  | 23 | -0.42 | -1.14 | 0.295 | 0.323 | 1.000 | 775 | tags=17%, list=9%, signal=19% |
| 44 | IVY\_LE |  | 24 | -0.41 | -1.13 | 0.290 | 0.331 | 1.000 | 629 | tags=21%, list=7%, signal=22% |
| 45 | WENG\_STEMNESS\_PMID\_30982771 |  | 34 | -0.38 | -1.12 | 0.296 | 0.346 | 1.000 | 802 | tags=21%, list=9%, signal=23% |
| 46 | RICHARDS\_LIDDELOW\_NATURE\_2017\_A2\_REACTIVE\_ASTROCYTES |  | 27 | -0.38 | -1.07 | 0.363 | 0.429 | 1.000 | 547 | tags=26%, list=6%, signal=28% |
| 47 | VANGRONINGEN\_ADRN |  | 223 | -0.27 | -1.07 | 0.310 | 0.435 | 1.000 | 1502 | tags=23%, list=17%, signal=27% |
| 48 | GANDAL\_AUTISM\_UP\_PMID29439242 |  | 80 | -0.30 | -1.05 | 0.364 | 0.476 | 1.000 | 1132 | tags=20%, list=13%, signal=23% |
| 49 | IVY\_CTPAN |  | 98 | -0.29 | -1.04 | 0.360 | 0.469 | 1.000 | 1007 | tags=17%, list=12%, signal=19% |
| 50 | ALMSTEDT\_17Q\_MSIGDB |  | 341 | -0.24 | -0.99 | 0.482 | 0.587 | 1.000 | 1755 | tags=22%, list=20%, signal=27% |
| 51 | ALMSTEDT\_11Q\_MSIGDB |  | 297 | -0.24 | -0.98 | 0.518 | 0.611 | 1.000 | 1432 | tags=19%, list=16%, signal=21% |
| 52 | DOROSZKO\_U3180MG\_CLUSTER\_6 |  | 39 | -0.31 | -0.95 | 0.528 | 0.664 | 1.000 | 2174 | tags=46%, list=25%, signal=61% |
| 53 | GAROFANO\_IAVARONE\_LIST\_OF\_NEURAL\_PROGENITOR\_MARKER\_GENES |  | 79 | -0.27 | -0.94 | 0.556 | 0.689 | 1.000 | 377 | tags=10%, list=4%, signal=10% |
| 54 | ALMSTEDT\_RISK |  | 30 | -0.31 | -0.90 | 0.623 | 0.768 | 1.000 | 1171 | tags=27%, list=13%, signal=31% |
| 55 | GANDAL\_DEPRESSION\_DN\_PMID29439242 |  | 31 | -0.30 | -0.85 | 0.719 | 0.870 | 1.000 | 2184 | tags=35%, list=25%, signal=47% |
| 56 | RICHARDS\_CAHOY\_JNEUROSCI\_2011\_ASTRO\_MATURE |  | 467 | -0.20 | -0.83 | 0.950 | 0.902 | 1.000 | 1837 | tags=21%, list=21%, signal=25% |
| 57 | DOROSZKO\_U3054MG\_CLUSTER\_10 |  | 235 | -0.21 | -0.82 | 0.893 | 0.895 | 1.000 | 1943 | tags=28%, list=22%, signal=35% |
| 58 | ROSEN\_KRONA\_TI\_UNPUBL |  | 46 | -0.26 | -0.81 | 0.780 | 0.905 | 1.000 | 1506 | tags=22%, list=17%, signal=26% |
| 59 | NB\_KI\_CLUSTER13 |  | 110 | -0.22 | -0.81 | 0.851 | 0.894 | 1.000 | 986 | tags=14%, list=11%, signal=15% |
| 60 | NB\_KI\_CLUSTER12 |  | 35 | -0.27 | -0.80 | 0.788 | 0.885 | 1.000 | 880 | tags=14%, list=10%, signal=16% |
| 61 | SUVA\_G1S\_PMID\_31327527 |  | 26 | -0.25 | -0.71 | 0.880 | 0.966 | 1.000 | 1208 | tags=23%, list=14%, signal=27% |
| 62 | RICHARDS\_NEFTEL\_CELL\_2019\_G1\_S |  | 26 | -0.25 | -0.71 | 0.865 | 0.952 | 1.000 | 1208 | tags=23%, list=14%, signal=27% |
Table: Gene sets enriched in phenotype **na **[plain text format]****
